# Supplementary material for: A Pilot Study on the Effects of Transcranial Direct Current Stimulation on Brain Rhythms and Entropy during Self-Paced Finger Movement using the Epoc Helmet
Source: Front Hum Neurosci. 2017 Apr 25;11:201. doi: 10.3389/fnhum.2017.00201 (PMC5408787; doi:10.3389/fnhum.2017.00201)
Supplement: Supplementary file 1 [file Data_Sheet_1.zip › Complementary_results/Frequency_Bands_Complete_EEG/Theta/Theta_Band_Complete-EEG_F4.pdf]

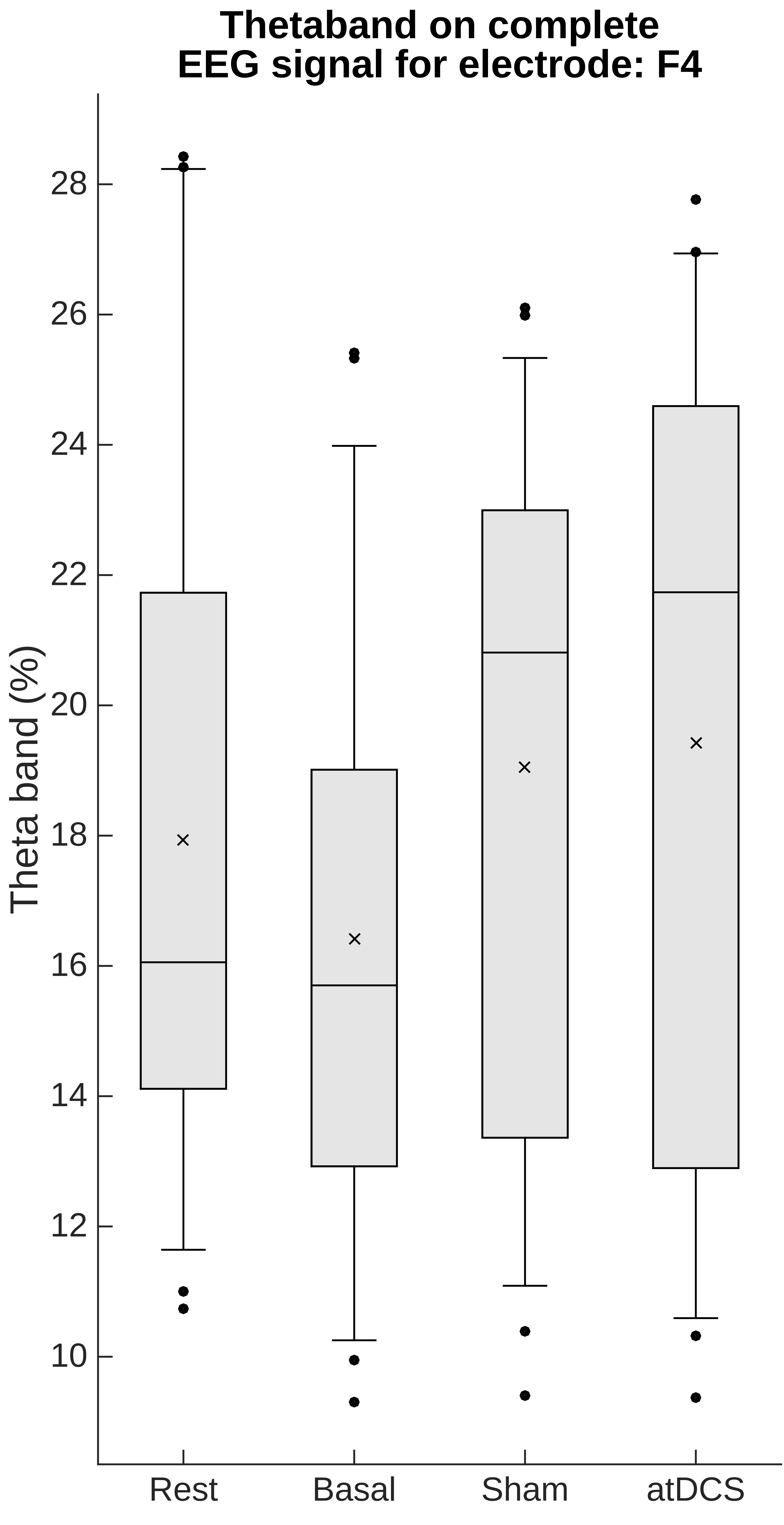
Thetaband on complete
EEG signal for electrode: F4
Theta band (%)
28
26
24
22
20
18
16
14
12
10
Rest
Basal
Sham
atDCS
